# Superconducting coherence length of hole-doped cuprates obtained from electron–boson spectral density function

**DOI:** 10.1038/s41598-021-91163-w

**Published:** 2021-06-03

**Authors:** Jungseek Hwang

**Affiliations:** Department of Physics, Sungkyunkwan University, Suwon, Gyeonggi-do 16419 Republic of Korea

**Keywords:** Physics, Condensed-matter physics, Superconducting properties and materials

## Abstract

Electron–boson spectral density functions (EBSDFs) can be obtained from measured spectra using various spectroscopic techniques, including optical spectroscopy. EBSDFs, known as glue functions, are suggested to have a magnetic origin. Here, we investigated EBSDFs obtained from the measured optical spectra of hole-doped cuprates with wide doping levels, from underdoped to overdoped cuprates. The average frequency of an EBSDF provides the timescale for the spin fluctuations to form Cooper pairs. This timescale is directly associated with retarded interactions between electrons. Using this timescale and Fermi velocity, a reasonable superconducting coherence length, which reflects the size of the Cooper pair, can be extracted. The obtained coherence lengths were consistent with those measured via other experimental techniques. Therefore, the formation of Cooper pairs in cuprates can be explained by spin fluctuations, the timescales of which appear in EBSDFs. Consequently, EBSDFs provide crucial information on the timescale of the microscopic mechanism of Cooper pair formation.

## Introduction

Since the discovery of copper-oxide superconductors (or cuprates)^1^, intensive experimental and theoretical studies have been performed on these intriguing material systems. The primary objectives of such studies included determining the microscopic pairing mechanism of the Cooper pairs in cuprate systems. Based on the generic phase of cuprates, antiferromagnetic spin fluctuations are expected to play an important role in the formation of Cooper pairs in such systems. One important result might be the electron–boson spectral density function (EBSDF) obtained from spectra measured using various spectroscopic techniques, including optical spectroscopy^2–13^. This function, in principle, describes the interaction of two electrons through the exchange of force-mediating bosons; thus, the EBSDF has also been called the glue function^10^. The experimentally obtained EBSDFs are suggested to have a magnetic origin, that is, antiferromagnetic spin fluctuations^2–4,8,12,14–18^. However, the issue on the origin of the EBSDFs is still debating. This quantity can be denoted as $I^2B(\omega )$, where *I* is the couple constant between the electron and the boson and $B(\omega )$ is the boson spectrum. Therefore, this glue function may carry crucial information on the formation of Cooper pairs. EBSDFs can be extracted from measured reflectance spectra using a well-developed procedure^3,4,8,10,19,20^. From the obtained EBSDFs, the coupling constants and the superconducting transition temperatures ($T_c$) can be determined using a generalized McMillan formula^10,11,21^. Because the maximum possible* T*_c_ obtained from the experimental EBSDFs using the McMillan formula are higher than the actual superconducting transition temperatures, EBSDFs may be sufficient for realizing superconductivity. However, the microscopic mechanism of Cooper pair formation in cuprates has not yet been elucidated. Therefore, determining the microscopic paring mechanism has been an important and debated issue in contemporary condensed matter physics.

In this study, we demonstrate that the superconducting coherence length can be obtained from the experimental EBSDFs of cuprates at various doping levels (from underdoped to overdoped). The experimental EBSDFs were obtained from the measured optical spectra of Bi$_2$Sr$_2$CaCu$_2$O$_{8+\delta }$ (Bi-2212) and YBa$_2$Cu$_3$O$_{6.50}$ (Y123-orthoII). First, we obtained the average frequency of the EBSDF, which provides the timescale for the retarded interaction between two electrons in a Cooper pair. The coherence length is directly related to the size of the Cooper pair, and is determined by the Fermi velocity and timescale obtained from the experimental EBSDF. We also considered both the two-dimensional transport character and the anisotropic (*d*-wave) superconducting gap to obtain the *ab*-plane coherence length. The resulting coherence lengths at various doping levels are reasonable, compared with the coherence lengths measured using other experimental techniques.

Based on this study, we propose a microscopic pairing mechanism that is conceptually similar to that of conventional Bardeen–Cooper–Schrieffer (BCS) superconductors. However, there are some differences between the two superconductor types. The force-mediating boson is associated with spin fluctuations above the background (antiferromagnetic) spin in cuprates, whereas the boson is associated with the charge fluctuations above the background (lattice ion) charge in BCS superconductors. We assume that the spin fluctuations in cuprates play the same role as charge fluctuations in BCS superconductors. Therefore, a retarded interaction is the force required to form Cooper pairs in cuprates. The coherence lengths of cuprates are much shorter (usually two orders of magnitude) than those of BCS superconductors. This can be understood by considering that spin fluctuations have much shorter timescales compared with those of charge fluctuations, and the Fermi velocities of cuprates are slower than those of BCS superconductors.

## Method and results

### Conventional superconductor, Pb

**Figure 1:**
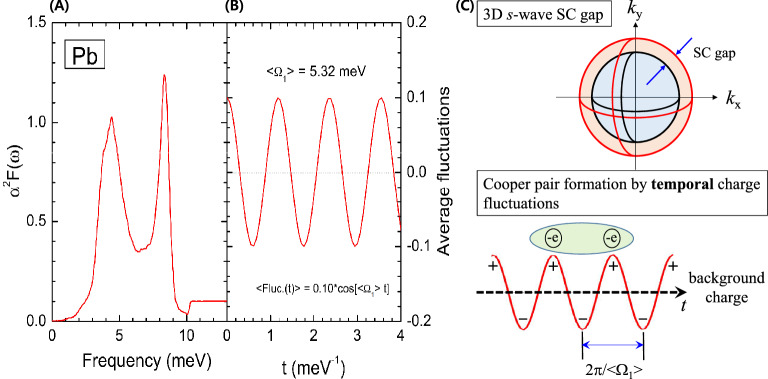
A conventional Bardeen–Cooper–Schrieffer (BCS) superconductor, Pb. (**A**) Electron–phonon spectral density function ($\alpha ^2 F(\omega )$) of Pb. (**B**) Average charge fluctuations on top of the regular ion background charge. (**C**) Schematic diagrams for the *s*-wave superconducting (SC) gap and the Copper pair formation by the average charge fluctuations. Here $+$ and − represent the positive and negative excess charge above the background charge, respectively.

Let us first consider a conventional BCS superconductor, Pb, which has been thoroughly studied. The electron–phonon spectral density function (EPSDF), $\alpha ^2F(\omega )$, of Pb was obtained using theoretical calculations and experimental spectroscopic techniques, including optical spectroscopy^22–25^. The obtained EPSDF of Pb is shown in Fig. 1A. The EPSDF of Pb was used in this study to estimate the superconducting coherence length. First, the average frequency ($\langle \Omega _1\rangle$) of the EPSDF was calculated, defined as $\langle \Omega _1\rangle \equiv (2/\lambda )\int _{0}^{\omega _c} [\alpha ^2F(\omega )/\omega ] \omega d\omega$, where $\lambda$ is the coupling constant given by $\lambda \equiv 2\int _{0}^{\omega _c} [\alpha ^2F(\omega )/\omega ] d\omega$, with a cutoff frequency of $\omega _c$. We note that the EPSDFs of conventional superconductors, including Pb, may exhibit some temperature-dependence. However, $\lambda$ does not depend on the temperature. The inverse of the average frequency ($2\pi /\langle \Omega _1\rangle$) is the average period of charge fluctuations in the EPSDF. The average charge fluctuation ($Fluc_{charge}(t)$) is described as $Fluc_{charge} = 0.10 \cos (\langle \Omega _1\rangle t)$. Note that the amplitude of the fluctuation is arbitrary. The fluctuating charge with the average frequency is shown in Fig. 1B. The period of the fluctuating charge can be the timescale for the retarded interaction between two electrons in a Cooper pair. In Fig. 1C a schematic of the s-wave superconducting gap and Cooper pair formation by the *temporal* charge fluctuations is presented. As electrons have negative charge, two electrons can be paired at the maxima of the positive charge of the temporal fluctuations through the retarded interaction. The Coulomb repulsion between electrons in a pair can be overcome through this retarded attractive electric interaction. The coherence length ($\xi$) can be obtained using the timescale ($2\pi /\langle \Omega _1\rangle$) and the Fermi velocity ($v_{F}$). Because conventional BCS superconductors have three-dimensional (isotropic) *s*-wave gaps, an electron can be paired with any other electron on a sphere with the electron at the center. Consequently, spatial degeneracy increases the pairing probability by a factor of 4$\pi$, which is the full solid angle in three-dimensional space. This results in a coherence length that is shorter by the same factor. Therefore, the coherence length can be written as $\xi = v_F [2\pi /\langle \Omega _1\rangle ](1/4\pi ) = v_F/(2\langle \Omega _1\rangle )$. The Fermi velocity of Pb is $\sim 1.83 \times 10^{6}$ m/s^26,27^. The estimated coherence length is 113 nm, which is consistent with the reported value of 96 nm^28^. Therefore, this approach is adequate for estimating the coherence length of a conventional superconductor, Pb. Further, this picture shows that Cooper pairs can be formed by a combination of the electron velocity at the Fermi surface and the timescale of the charge fluctuations. This explains why the Cooper pairs have much larger sizes compared with the spacings between electrons in the material system. Using this approach, we demonstrated that one of two characteristic length scales (coherence length and London penetration depth) for superconductivity can be determined from the EPSDF. Note that the EPSDF clearly carries information on the timescale of the retarded interaction.

### Unconventional superconductors, cuprates

**Figure 2:**
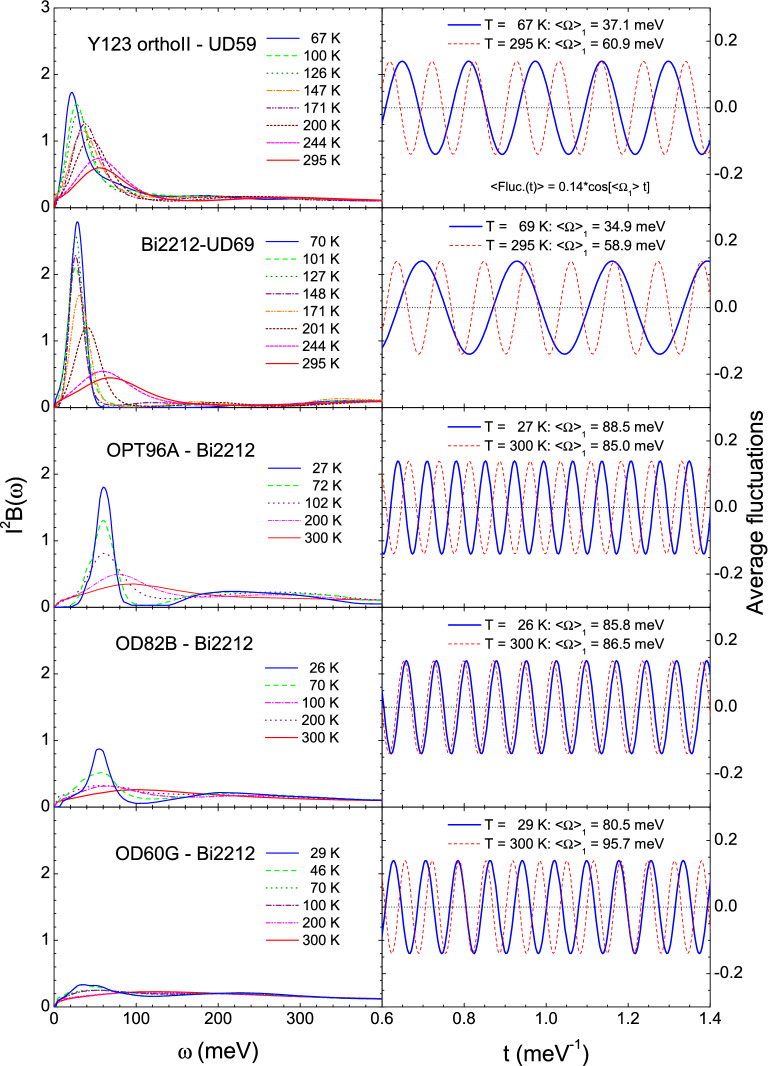
Electron–boson spectral density functions (EBSDFs) of cuprates and their average frequencies. (Left column) EBSDFs ($I^2B(\omega )$) of cuprates at various doping levels and temperatures. (Right column) Average fluctuating spins of cuprates with the average frequencies at two (lowest and room) temperatures.

**Figure 3:**
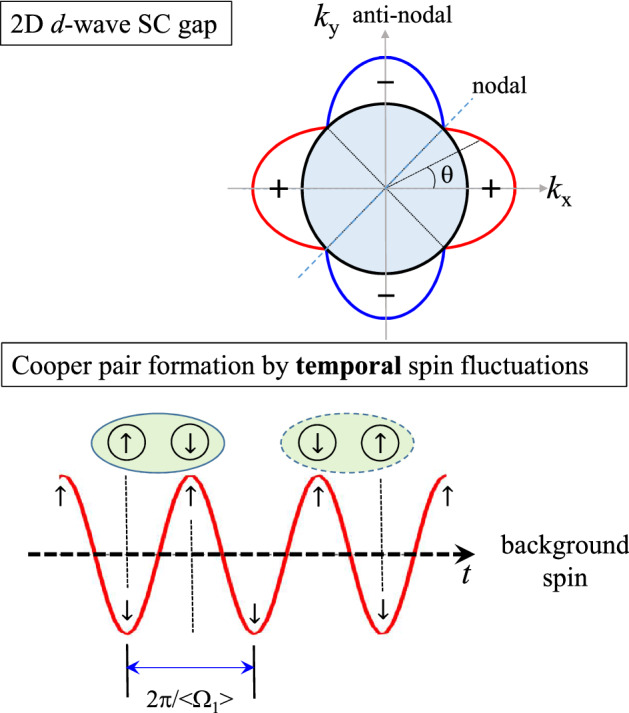
High-transition-temperature superconductors, cuprates. (Upper) Schematic of the d-wave superconducting (SC) gap. (Lower) Cooper pair formation by the average spin fluctuations. Here, $\uparrow$ and $\downarrow$ indicate the up and down excess spins above the background spin, respectively.

**Figure 4:**
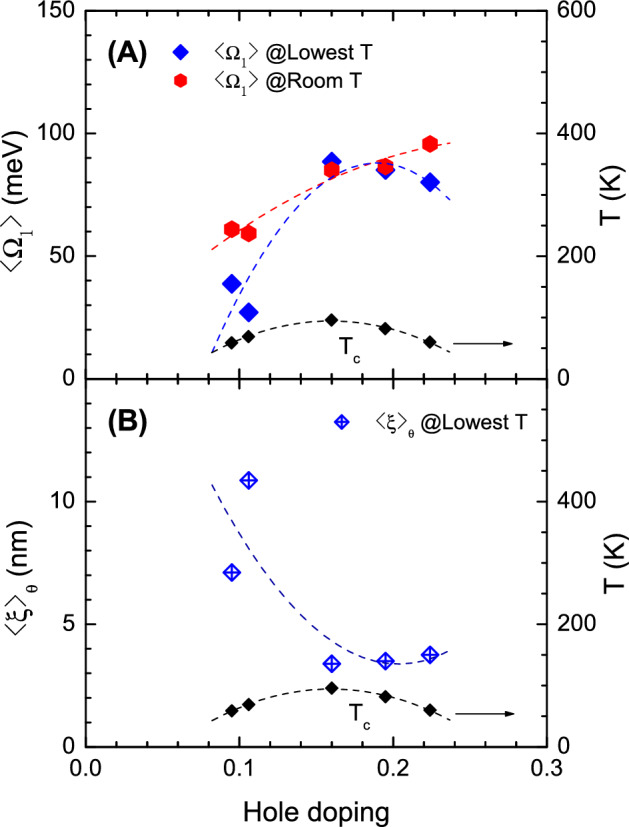
Average frequencies of $I^2 B(\omega )$ and the coherence lengths for cuprates at various doping levels. (**A**) The doping-dependent average frequencies at two (lowest and room) temperatures. (**B**) The doping-dependent coherence lengths at the lowest temperature in the superconducting state.

We applied the previously described approach to cuprate systems. We used the EBSDFs of cuprates obtained from optically measured spectra^29,30^. The EBSDFs of YBa$_2$Cu$_3$O$_{6.50}$ orthoII (Y123 orthoII) with $T_c = 59$ K at various temperatures are obtained by using a maximum entropy method^30,31^ (also see “Supplementary Materials”). The EBSDFs obtained for Y123-orthoII and Bi$_2$Sr$_2$CaCu$_2$O$_{8+\delta }$ (Bi-2212) at various doping levels and temperatures are shown in the left column of Fig. 2. The optically determined EBSDFs have been suggested to have a magnetic origin because the doping- and temperature-dependent behaviors of the EBSDFs are consistent with the spectrum of spin fluctuation as measured by inelastic neutron scattering^2–4,8,12,16,17^. Therefore, we assumed that they are associated with the antiferromagnetic spin fluctuations. In the right column of Fig. 2, the fluctuating spins are presented with the average frequencies of the EBSDFs above the background spin at two temperatures (lowest and ambient) for each sample. The average spin fluctuation ($Fluc_{spin}(t)$) is $Fluc_{spin} = 0.14 \cos (\langle \Omega _1\rangle t)$. Note that the amplitude of the fluctuation is arbitrary. For the optimally doped sample, the average frequency at the lowest temperature was slightly higher than that at room temperature, while the results for the underdoped and overdoped samples were the opposite (see Fig. 4A). The temperature-dependent average frequencies for all samples can be found in the “Supplementary Materials”. The coherence length can be obtained using a similar approach to that described previously for BCS superconductors. However, there are some differences between BCS superconductors and cuprates. In cuprates, the charge transport exhibits strong anisotropy; the *ab* plane transport is dominant to that along the *c* axis^32^. Superconductivity typically occurs in the two-dimensional CuO$_2$ (or *ab*) plane. Additionally, cuprates have anisotropic *d*-wave superconducting gaps. The timescale of the retarded interaction in the spin fluctuation is reduced by a factor of 2 because two possible singlet pairs can be formed with a timescale of half the full period ($2\pi /\langle \Omega _1 \rangle$), as shown in the lower part of Fig. 3. The binding force for Cooper pair formation is directly related to the retarded interaction caused by the spin fluctuations. The retarded interaction is most likely associated with an attractive spin–spin (or spin-up and spin-down) interaction. Because of these crucial differences, the coherence length formula used for BCS superconductors should be modified. The first modification is associated with the two-dimensionality. An electron can be paired with any other electron on a circle with the electron at the center; thus, the coherence length can be reduced by a factor of $2\pi$, which is a full angle in two dimensions. The second modification is associated with the anisotropic *d*-wave superconducting gap, specifically, $\Delta (\theta ) = \Delta _0 \cos (2\theta )$, where $\Delta _0$ is the maximum gap along the anti-nodal direction and the angle ($\theta$) is measured from the anti-nodal direction as shown in the upper part of Fig. 3. In general, the coherence length is inversely proportional to the superconducting gap^33^. Therefore, the coherence length is dependent on $\theta$, that is, $\xi (\theta )$. In this case, we need to average the coherence length over the angle in [0, $\pi /4$], as $\langle \xi \rangle _{\theta } \equiv (4/\pi )\int _{0}^{\pi /4} \xi (\theta ) d\theta$. The angle-dependent coherence length can be written as, $\xi (\theta ) = v_F(1/2)[2\pi /\langle \Omega _1\rangle ](1/2\pi )(1/\cos (2\theta ))$. Here, 1/2 comes from the timescale reduction by a factor of 2. The average coherence length can be written as $\langle \xi \rangle _{\theta }= v_F/(2\langle \Omega _1\rangle ) \langle 1/\cos (2\theta )\rangle _{\theta }$. Here, $\langle 1/\cos (2\theta )\rangle _{\theta } =(2/\pi ) \ln [1/\cos (2\theta )+\tan (2\theta )]$, which shows a logarithmic singularity along the nodal direction (or at $\theta = \pi /4$). To estimate the average coherence length, we assumed that $\langle 1/\cos (2\theta )\rangle _{\theta }\cong$ 3.33. In this case, we used $\pi /4 \cong 0.78$ rad. Although some uncertainties exist in determining the experimentally reliable value of $\langle 1/\cos (2\theta )\rangle _{\theta }$ the assumption might be reasonable if the size of the maximum superconducting gap and the experimental accessibility to the lowest frequency cutoff are considered; if $\Delta _0 =$ 30 meV, $\Delta$(0.78 rad) = 0.32 meV (or 2.6 cm^-1^). In general, optical spectra exclude data below approximately 50 cm^-1^, which is well above the $\Delta$(0.78 rad).

The average coherence lengths of the cuprate samples were estimated at various doping levels and at two (lowest and room) temperatures using the modified formula for cuprates, as discussed previously: $\langle \xi \rangle _{\theta }= v_F/(2\langle \Omega _1\rangle )\times 3.33$. The Fermi velocity of Bi-2212 is approximately $2.7 \times 10^5$ m/s^34^. The same Fermi velocity value was used for all B-2212 samples. Meanwhile, the Fermi velocity of the Y123-orthoII sample is approximately $2.5\times 10^5$ m/s^35^. The average frequencies of $I^2B(\omega )$ at the two temperatures are shown in Fig. 4A. As aforementioned, the average frequency at the lowest temperature was higher than that at room temperature only for the optimally doped sample. The corresponding estimated average coherence lengths at the lowest temperature are shown in Fig. 4B. These results exhibit a strong doping dependence; the near-optimally doped coherence length is the shortest at approximately 3.5 nm. The estimated coherence lengths are reasonable compared with reported values; for example, $\xi _{ab} \simeq 1.6$ nm for an optimally doped YBa$_2$Cu$_3$O$_{6.90}$ with $T_c =$ 95 K and $\xi _{ab} =3.8$ nm for optimally doped La$_{1.85}$Sr$_{0.16}$CuO$_4$ with $T_c =38$ K^36^. In general, the coherence lengths of conventional BCS superconductors are hundreds of nanometers, whereas those of cuprates are in the nanometer range^37^. It should be noted that these estimated coherence lengths might be overestimates because our lowest cutoff frequency ($\sim 50$ cm^-1^) is much higher than the one estimated ($\Delta$(0.78 rad)$\cong$ 2.6 cm^-1^ for $\Delta _0 = 30$ meV).

## Summary and conclusion

**Figure 5:**
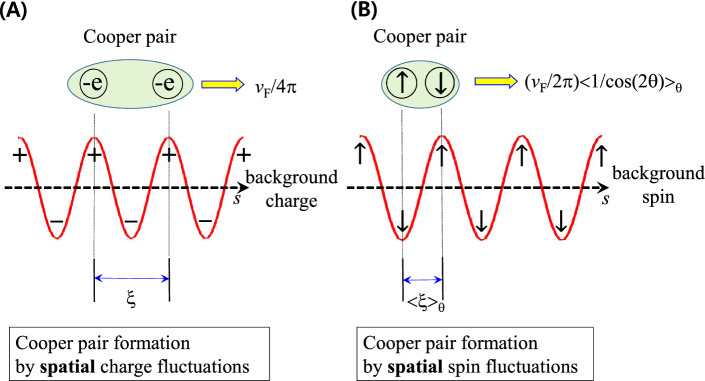
*Spatial* charge/spin fluctuations and Cooper pair formations. (**A**) Cooper pair formation by electric retarded interaction caused by the charge fluctuations in the conventional BCS superconductors. (**B**) Cooper pair formation by the retarded interaction caused by the spin fluctuations in the cuprate systems.

The Cooper pair formations are summarized through the spin (or charge) fluctuations in Fig. 5. The charge/spin fluctuations are the bonding glue for the formation of Cooper pairs moving with effective Fermi velocities, as shown in Fig. 5A,B. The size of the Cooper pair (or coherence length) should adequately match the average period of *spatial* spin (or charge) fluctuations. The high-temperature superconductivity can be understood through the microscopic mechanism, in which spin-1 bosons with an antiferromagnetic spin texture are involved. The coherence lengths obtained from the experimental EBSDFs are consistent with those obtained from other experiments. The concept of the microscopic mechanism of Cooper pair formation in cuprates is similar to that in conventional BCS superconductors, except for some detailed differences. The first is in the force-mediating boson, which is associated with the spin fluctuations instead of the charge fluctuations in BCS superconductors. It is worth to be noted that recent resonance inelastic X-ray scattering studies on cuprates have shown multiorbital charge density wave excitations and their association with electron-phonon anomalies^38,39^, indicating that the charge fluctuations may not be completely ruled out. Other differences include the shorter timescale of spin fluctuations by one order and slower Fermi velocities by one order compared with the corresponding quantities for conventional superconductors. These differences result in shorter coherence lengths of cuprates by two orders compared with those of BCS superconductors.

One interesting question might be how the microscopic pairing information is encoded in the measured optical spectra. The optical spectra show the optical transitions averaged over the *k*-space, which are the transitions of electrons from occupied states below the Fermi level to unoccupied states above it. When the bands are renormalized by correlations between electrons caused by many-body effects, the band renormalization effects will be naturally encoded in the measured spectrum indirectly in the case of spin fluctuations. From an appropriate analysis (or decoding) process, the correlation spectrum can be extracted from the measured spectrum, which is the EBSDF spectrum. The coherence length obtained from the timescale in the extracted EBSDF spectrum and the Fermi velocity is reasonable. In addition, the superconducting transition temperatures estimated from the EBSDF using a generalized McMillan formula are acceptable^11,21^. Therefore, the EBSDF may provide conclusive evidence for high-temperature superconductivity. In particular, using optical spectroscopy, the EBSDFs of almost all cuprates can be obtained because optical spectroscopy is sensitive to small signals, provides bulk properties, and covers a wide spectral range with a high energy resolution. Therefore, it can be a bridge between other experimental techniques, such as angle-resolved photoemission spectroscopy (surface sensitive) and inelastic neutron scattering (small scattering cross section). We expect that this approach can be applied to obtain the coherence lengths from EBSDFs established by other experimental techniques. We hope that our results will help researchers to conclusively establish the microscopic pairing mechanism for high-temperature superconductors, including cuprates and Fe-based superconductors.

## Supplementary information

Supplementary Information.

## References

[1] Bednorz TG, Muller A (1986). Possible high T_c_ superconductivity in the Ba–La–Cu–O system. Z. Phys. B.

[2] Carbotte JP, Schachinger E, Basov DN (1999). Coupling strength of charge carriers to spin fluctuations in high-temperature superconductors. Nature (London).

[3] Schachinger E, Carbotte JP (2000). Coupling to spin fluctuations from conductivity scattering rates. Phys. Rev. B.

[4] Hwang J (2006). a-axis optical conductivity of detwinned ortho-II YBa_2_Cu_3_O_6.50_. Phys. Rev. B.

[5] Zasadzinski JF (2006). Persistence of strong electron coupling to a narrow boson spectrum in overdoped Bi_2_Sr_2_CaCu_2_O_8+δ_ tunneling data. Phys. Rev. Lett..

[6] Lee J (2006). Interplay of electron–lattice interactions and superconductivity in Bi_2_Sr_2_CaCu_2_O_8+δ_. Nature (London).

[7] Valla T (2007). High-energy kink observed in the electron dispersion of high-temperature cuprate superconductors. Phys. Rev. Lett..

[8] Hwang J, Timusk T, Schachinger E, Carbotte JP (2007). Evolution of the bosonic spectral density of the high-temperature superconductor Bi_2_Sr_2_CaCu_2_O_8+δ_. Phys. Rev. B.

[9] Hwang J, Timusk T, Carbotte JP (2007). Scanning-tunnelling spectra of cuprates. Nature (London).

[10] van Heumen E (2009). Optical determination of the relation between the electron-boson coupling function and the critical temperature in high-Tc cuprates. Phys. Rev. B.

[11] Hwang J (2011). Electron-boson spectral density function of underdoped Bi_2_Sr_2_CaCu_2_O_8+δ_ and YBa_2_Cu_3_O_6.50_. Phys. Rev. B.

[12] Carbotte JP, Timusk T, Hwang J (2011). Bosons in high-temperature superconductors: An experimental survey. Rep. Progress Phys..

[13] Ahmadi O, Coffey L, Zasadzinski JF, Miyakawa N, Ozyuzer L (2011). Eliashberg analysis of tunneling experiments: Support for the pairing glue hypothesis in cuprate superconductors. Phys. Rev. Lett..

[14] Johnson PD (2001). Doping and temperature dependence of the mass enhancement observed in the cuprate Bi_2_Sr_2_CaCu_2_O_8+δ_. Phys. Rev. Lett..

[15] Zasadzinski JF (2001). Correlation of tunneling spectra in Bi_2_Sr_2_CaCu_2_O_8+δ_ with the resonance spin excitation. Phys. Rev. Lett..

[16] Hwang J, Timusk T, Gu GD (2004). High-transition-temperature superconductivity in the absence of the magnetic-resonance mode. Nature (London).

[17] Norman M (2004). Shine a light. Nature (London).

[18] Dahm T (2009). Strength of the spin-fluctuation-mediated pairing interaction in a high-temperature superconductor. Nat. Phys..

[19] van Heumen E, Meevasana W, Kuzmenko AB, Eisaki H, van der Marel D (2009). Doping-dependent optical properties of Bi2201. New J. Phys..

[20] Hwang J (2015). Reverse process of usual optical analysis of boson-exchange superconductors: Impurity effects on s- and d-wave superconductors. J. Phys. Condens. Matter.

[21] Hwang J (2008). Bosonic spectral density of epitaxial thin-film La_1.83_Sr_0.17_CuO_4_ superconductors from infrared conductivity measurements. Phys. Rev. Lett..

[22] McMillan WL, Rowell JM (1965). Lead phonon spectrum calculated from superconducting density of states. Phys. Rev. Lett..

[23] Farnworth B, Timusk T (1974). Far-infrared measurements of the phonon density of states of superconducting lead. Phys. Rev. B.

[24] Farnworth B, Timusk T (1976). Phonon density of states of superconducting lead. Phys. Rev. B.

[25] Tomlinson PG, Carbotte JP (1976). Anisotropic superconducting energy gap in Pb. Phys. Rev. B.

[26] Lykken GI, Geiger AL, Dy KS, Mitchell EN (1971). Measurement of the superconducting energy gap and fermi velocity in single-crystal lead films by electron tunneling. Phys. Rev. B.

[27] Ashcroft NW, Mermin ND (1976). Solid State Physics.

[28] Gasparovic RF, McLean WL (1970). Supercondueting penetration depth of lead. Phys. Rev. B.

[29] Hwang J, Carbotte JP, Timusk T (2008). Evidence for a pseudogap in underdoped Bi2Sr2CaCu2O8+δ and YBa2Cu3O6._50_ from in-plane optical conductivity measurements. Phys. Rev. Lett..

[30] Hwang J (2016). Intrinsic temperature-dependent evolutions in the electron-boson spectral density obtained from optical data. Sci. Rep..

[31] Schachinger E, Neuber D, Carbotte JP (2006). Inversion techniques for optical conductivity data. Phys. Rev. B.

[32] Ono S, Ando Y (2003). Evolution of the resistivity anisotropy in Bi_2_Sr_2−__*x*_La_*x*_CuO_6+δ_ single crystals for a wide range of hole doping. Phys. Rev. B.

[33] Tinkham M (1975). Introduction to Superconductivity.

[34] Vishik IM (2010). Doping-dependent nodal fermi velocity of the high-temperature super-conductor Bi2Sr2CaCu2O_–8+__δ_ revealed using high-resolution angle-resolved photoemission spectroscopy. Phys. Rev. Lett..

[35] Chiao M (2000). Low-energy quasiparticles in cuprate superconductors: A quantitative analysis. Phys. Rev. B.

[36] Mourachkine A (2002). High-Temperature Superconductivity in Cuprates: The Nonlinear Mechanism and Tunneling Measurements.

[37] Shen KM, Davis JCS (2008). Cuprate high-T_c_ superconductors. Mater. Today.

[38] Li J (2020). Multiorbital charge-density wave excitations and concomitant phonon anomalies in Bi_2_Sr_2_LaCuO_6+δ_. PNAS.

[39] Lin JQ (2020). Strongly correlated charge density wave in La2–*x*Sr*x*CuO4 evidenced by doping-dependent phonon anomaly. Phys. Rev. Lett..

