# Supplementary material for: Superconducting coherence length of hole-doped cuprates obtained from electron–boson spectral density function
Source: Sci Rep. 2021 Jun 3;11:11668. doi: 10.1038/s41598-021-91163-w (PMC8175355; doi:10.1038/s41598-021-91163-w)
Supplement: Supplementary file 1 — Supplementary Information. [file 41598_2021_91163_MOESM1_ESM.pdf]

Supplementary Materials

Superconducting coherence length of hole-doped cuprates obtained from electron-boson spectral density function

Jungseek Hwang

Department of Physics, Sungkyunkwan University, Suwon, Gyeonggi-do 16419, Republic of Korea

The electron-boson spectral density functions (EBSDFs) of Y123-orthoII UD 59 and B-2212 UD69

We used the maximum entropy method [S1] to obtain the EBSDFs of an underdoped Y123-orthoII with T_c = 59 K and an underdoped Bi-2212 with T_c = 69 K from existing measured optical scattering rates [S2, S3]. The EBSDFs of the underdoped Bi-2212 have been published [S3]. The data and fits of the two samples are shown in Fig. S1. The obtained EBSDFs show strong temperature-dependences, which have been proven as intrinsic properties [S3]. We used these EBSDFs for this study. The detailed description of the analysis method and the pseudogap model can be found in literatures [S1, S3, S4].

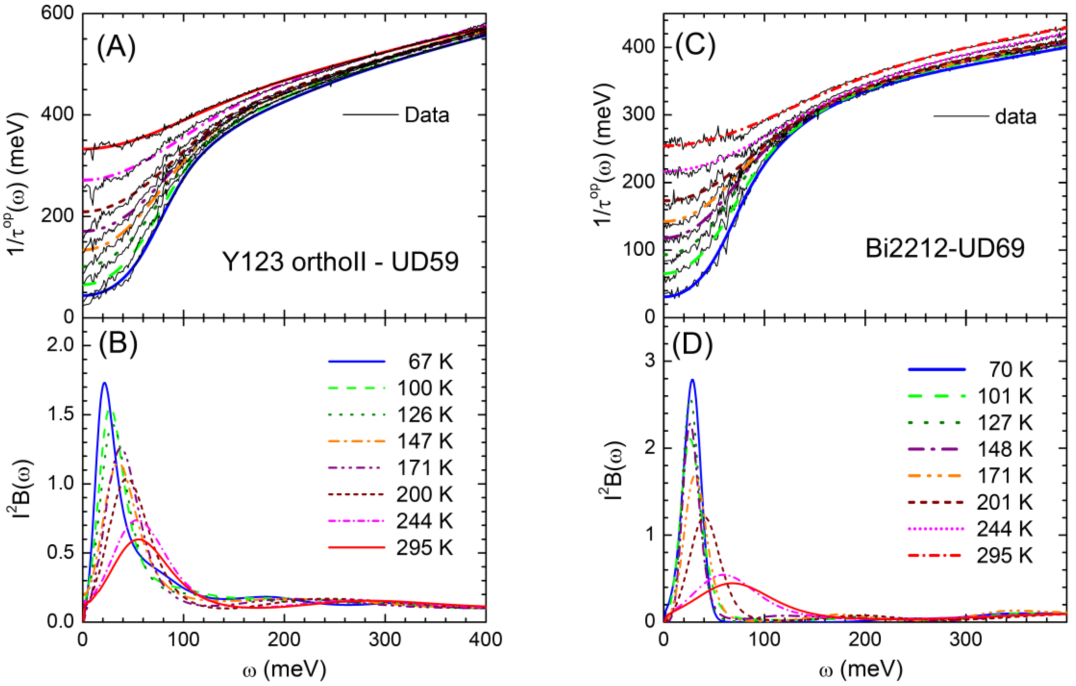

Figure S1. (A, B) Y123-orthoII UD 59 data and fits. (C, D) Bi-2212 UD 69 data and fits using the maximum entropy method including a pseudogap model [S1, S3].

Doping-dependent optical mode frequency, $\Omega_{op}(p)$ and coupling constant, $\lambda(p)$

In general, the EBSDF consists of a sharp peak and broad background [S5] at low temperature. The sharp peak is known as an optical (coherence) mode. We obtained the position of the sharp peak in the EBSDF at the lowest temperature and showed the doping-dependent peak position in Fig. S2(A). We also obtained the coupling constant, which is defined as $\lambda \equiv 2\int_0^{\omega_c}[I^2B(\omega)/\omega]\,d\omega$, and showed the doping-dependent coupling constants at two (lowest and room) temperatures in Fig. S2(B). The peak energy shows a maximum near optimally doped and roughly follows $7.2k_BT_c$ as shown in the figure. The coupling constant seems to be diverged in low doping region as shown in Fig. S2(B).

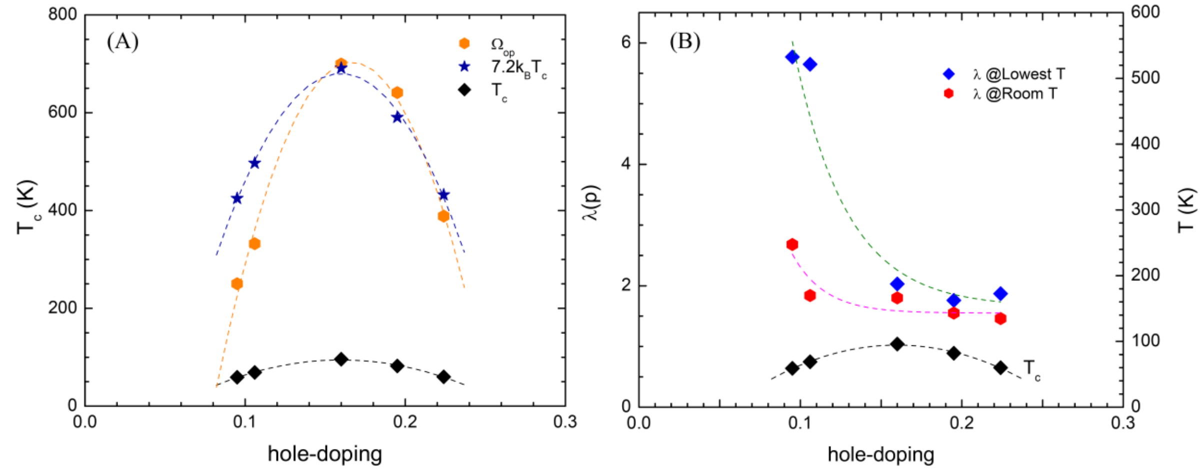

Figure S2. (A) Doping-dependent position of the optical mode. (B) The doping-dependent coupling constant (λ) at two (lowest and room) temperatures.

Temperature-dependent average frequencies of EBSDF, $\langle\Omega_1\rangle$, and average coherence length, $\langle\xi\rangle_\theta$ of cuprates

We obtained and showed the temperature-dependent coupling constant, average frequency of EBSDF, and the coherence length for each sample in Fig. S3(A), S3(B), and S3(C), respectively. They clearly show strong temperature- and doping-dependencies. As lowering the temperature, both the coupling constant and the coherence length increase but the average frequency slightly decreases. Underdoped samples show stronger temperature dependence. We note that the pseudogap shifts the optical mode to lower frequency and makes the time scale longer and eventually results in longer coherence length. Therefore, if one does not include the pseudogap in the analysis model the resulting EBSDF will give shorter coherence length compared with the coherence length obtained here since we included the pseudogap in the analysis model [S2, S3, S6].

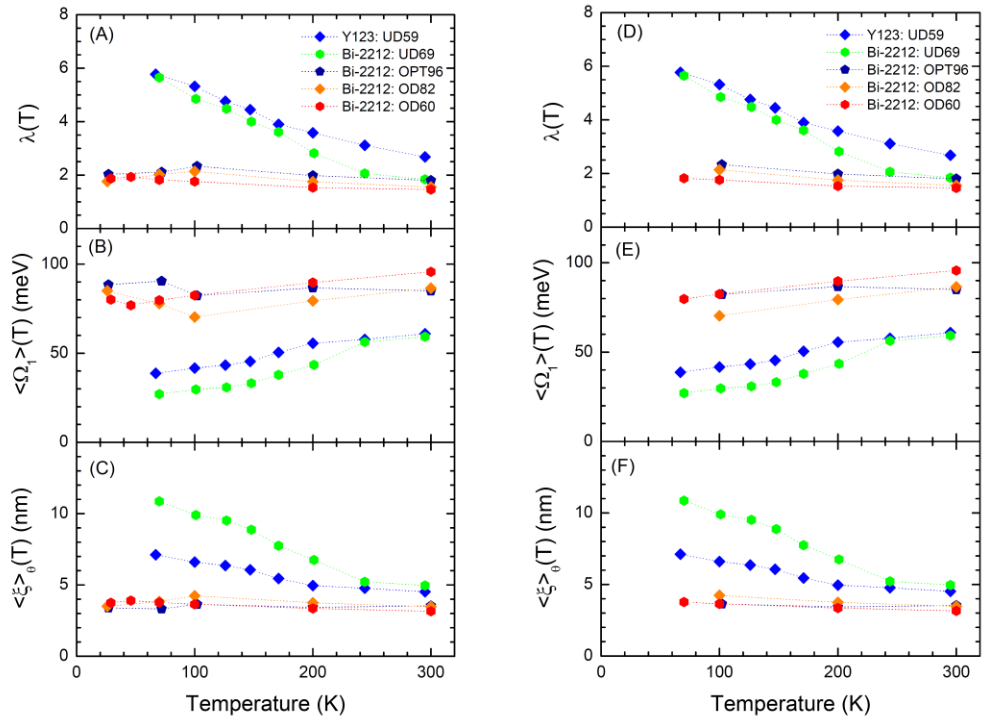

Figure S3. (Left) The temperature-dependent coupling constant (A), average frequency of EBSDF (B), and average coherence length (C) of cuprates at both normal and superconducting states for optimally and overdoped samples and at only normal state for underdoped samples. (Right) The temperature-dependent coupling constant (D), average frequency of EBSDF (E), and average coherence length (F) of cuprates at only normal state for the all samples.

Temperature-dependent logarithmically averaged frequencies, $\Omega_{\ln}$, and possible maximum T_c

One can obtain the superconducting transition temperature (T_c) from EBSDF using a generalized McMillan formula, $k_B T_c \cong 1.13 \frac{h}{2\pi} \Omega_{ln} \exp\left[-\frac{1+\lambda}{g\lambda}\right]$, where k_B is the Boltzmann constant, h is the Planck constant, λ is the coupling constant, $\Omega_{\ln}$ is the logarithmically averaged frequency of EBSDF defined as $\Omega_{\ln} \equiv \frac{1}{\lambda}\exp\left[2\int_0^{\omega_c} \frac{I^2B(\omega)}{\omega} (\ln\omega)\, d\omega\right]$, and g is an adjustable parameter ($0 < g \leq 1$), which oppose the superconductivity. When $g = 1$, T_c becomes maximum, T_c^{Max} [S6]. The obtained $\Omega_{\ln}$ and T_c^{Max} for the all samples are shown in Fig. S4.

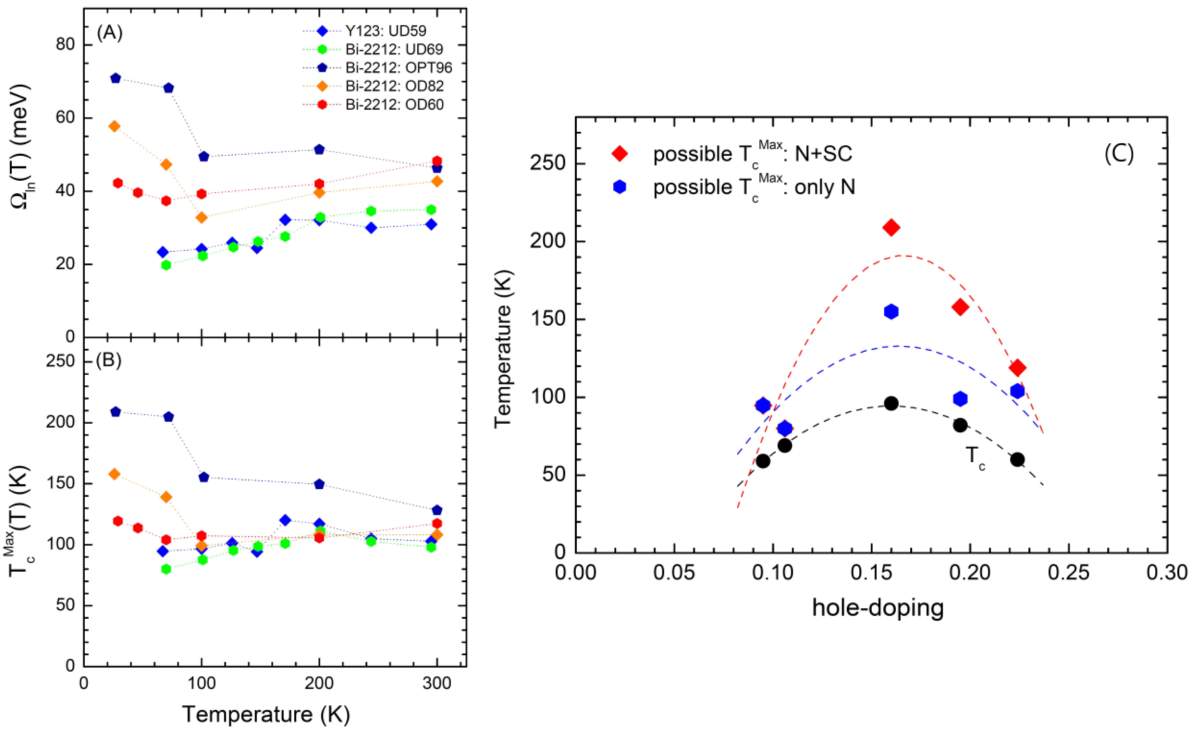

Figure S4. Temperature-dependent (A) logarithmically averaged frequency, $\Omega_{\ln}$ and (B) maximum possible superconducting temperature, T_c^{Max}, and (C) doping-dependent T_c^{Max} with using values at only normal state (blue hexagon) and also values using at normal state for underdoped samples and superconducting state for optimal and underdoped samples (red diamond).

Inverse Fourier transformations of EBSDFs

Here we performed the inverse Fourier transformation to get the time domain information of the EBSDFs. The results are shown in Fig. S5. We can observe a systematic change as the doping increases from underdoped to overdoped. In general, the EBSDF contains various frequency components as shown in the figure. We also show the (spin) fluctuation with the average frequency of EBSDF at the lowest temperature for each sample for comparison. The average frequency looks reasonable. In the underdoped samples, the sharp peak is dominant and its energy is similar to the average frequency. In the overdoped samples, the average frequency is higher than the sharp peak energy.

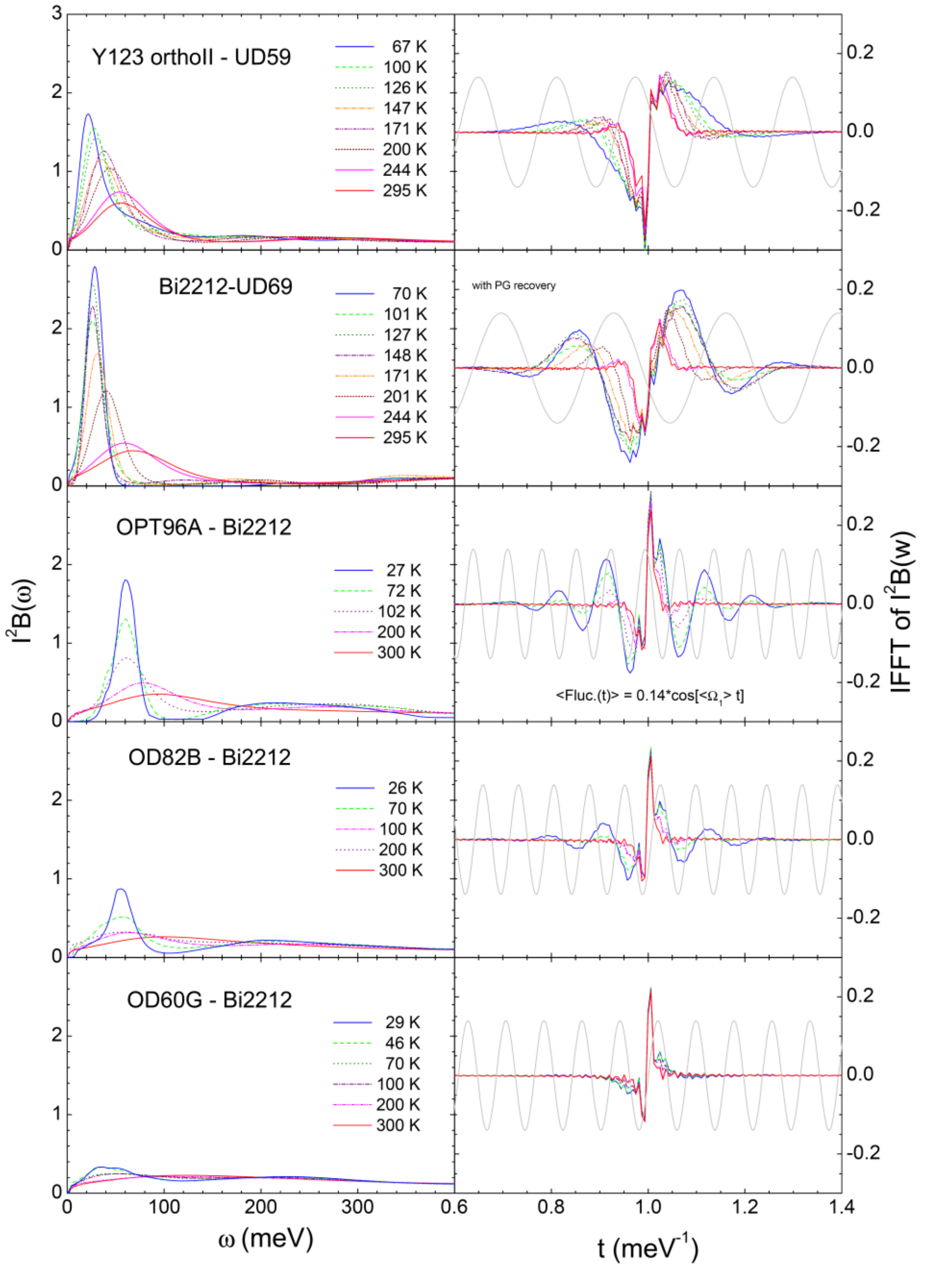

Figure S5. EBSDFs ($I^2B(\omega)$) and their corresponding inverse Fourier transforms at various doping levels and temperatures. The gray lines are the average oscillations (or fluctuations) of spin at the lowest temperatures above the background spin (see Fig. 3 in the main text).

References

[S1] E. Schachinger, D. Neuber, and J. P. Carbotte, Phys. Rev. B **73**, 184507 (2006).

[S2] J. Hwang, J. Yang, T. Timusk, S. G. Sharapov, J. P. Carbotte, D. A. Bonn, R. Liang, and W. N. Hardy, Phys. Rev. B **73**, 014508 (2006).

[S3] J. Hwang, Scientific Reports **6**, 23647 (2016).

[S4] J. Hwang, J. P. Carbotte, and T. Timusk, Euro. Phys. Lett. **82**, 27002 (2008).

[S5] J. P. Carbotte, T. Timusk, and J. Hwang, Reports on Progress in Physics **74**, 066501 (2011).

[S6] J. Hwang, Phys. Rev. B **83**, 014507 (2011).
